# Assessment of treatment adherence in liver transplant patients in relation to levels of depression, anxiety, disease acceptance and social support

**DOI:** 10.1192/j.eurpsy.2025.1366

**Published:** 2025-08-26

**Authors:** D. Schneider-Matyka, A. M. Cybulska, K. Rachubińska, M. Stanisławska, A. Kisielska, E. Grochans

**Affiliations:** 1 Department of Nursing; 2 Department of Infectious Diseases, Hepatology and Liver Transplantation, Pomeranian Medical University in Szczecin, Szczecin, Poland

## Abstract

**Introduction:**

Nonadherence in transplantology stands for a not cooperating patient in terms of taking medication, having the laboratory, and imaging tests taken, missing appointments, not following dietary and lifestyle recommendations related to smoking tobacco, drinking alcohol, and other high-risk behaviours. Factors of not following therapeutic recommendations in patient after liver transplantation are high costs, psychiatric disorders, convictions regarding harmfulness of the immunosuppressive medicine and its side effects as well as rejection episodes, infections, stress related to suffering from a chronic disease and inadequate social support.

**Objectives:**

The aim of this study was to assess the adherence to therapeutic recommendations in patients after liver transplantation including the level of depression, anxiety, degree of acceptance of the disease and social support.

**Methods:**

Study was carried out among 112 respondents after liver transplantation. The Delphi method was used and the following standardised tools were used: ISSB, AIS, BDI, STAI X-1 and X-2 as well as a questionnaire form regarding sociodemographic data.

**Results:**

An average level of adherence to therapeutic recommendations was on an average level (6.8±1.85). A statistically significant positive correlation between disease acceptance and following therapeutic recommendations was observed (r = –0.20, t = –2.040; p = 0.044). The adherence to recommendations increased with growing disease acceptance. Six factors were distinguished from the analysed predictors which in a relevant way influenced the level of adherence to therapeutic recommendations in the group of patients after liver transplantation. Regression model consisting of six independent variables explains 55% of variation of the dependent variable (r2corrected= 0,55, F(6, 98) = 22.127; p<0.001). Such positive factors include always following recommendations (β = –0.23; p = 0.002) and seeking various sources of information (β = –0.34; p<0.001), while negative ones constituted of illness duration (β = 0.18; p = 0.008), experiencing side effects (β = 0.40; p<0.001), suffering from concomitant diseases (β = 0,40; p<0,001).

**Conclusions:**

Patients who have accepted their disease constitute a group that adheres to therapeutic recommendations to a lesser extent.

The main factors affecting adherence to therapeutic recommendations are the search for other sources of information and declarative adherence to therapeutic recommendations. Negative predictors- duration of disease, experiencing adverse effects of treatment, and comorbidities.

The way in which a physician communicates to the patients after liver transplantation the information about the results indicating that the medications were taken irregularly significantly more often influenced not following therapeutic recommendations which might suggest an unintended non-adherence.

**Disclosure of Interest:**

None Declared

